# Liquid biopsies and cancer omics

**DOI:** 10.1038/s41420-020-00373-0

**Published:** 2020-11-26

**Authors:** Ivano Amelio, Riccardo Bertolo, Pierluigi Bove, Oreste Claudio Buonomo, Eleonora Candi, Marcello Chiocchi, Chiara Cipriani, Nicola Di Daniele, Carlo Ganini, Hartmut Juhl, Alessandro Mauriello, Carla Marani, John Marshall, Manuela Montanaro, Giampiero Palmieri, Mauro Piacentini, Giuseppe Sica, Manfredi Tesauro, Valentina Rovella, Giuseppe Tisone, Yufang Shi, Ying Wang, Gerry Melino

**Affiliations:** 1 Torvergata Oncoscience Research Centre of Excellence, TOR, Department of Experimental Medicine, University of Rome Tor Vergata, 00133 Rome, Italy; 2 School of Life Sciences, University of Nottingham, Nottingham, UK; 3 San Carlo di Nancy Hospital, Rome, Italy; 4 Indivumed GmbH, Hamburg, Germany; 5 Medstar Georgetown University Hospital, Georgetown University, Washington, DC USA; 6 CAS Key Laboratory of Tissue Microenvironment and Tumor, Shanghai Institute of Nutrition and Health, Shanghai Institutes for Biological Sciences, University of Chinese Academy of Sciences, Chinese Academy of Sciences, 320 Yueyang Road, 200031 Shanghai, China; 7 The First Affiliated Hospital of Soochow University and State Key Laboratory of Radiation Medicine and Protection, Institutes for Translational Medicine, Soochow University, 199 Renai Road, 215123 Suzhou, Jiangsu China

**Keywords:** Cancer genomics, Prognostic markers

## Abstract

The development of the sequencing technologies allowed the generation of huge amounts of molecular data from a single cancer specimen, allowing the clinical oncology to enter the era of the precision medicine. This massive amount of data is highlighting new details on cancer pathogenesis but still relies on tissue biopsies, which are unable to capture the dynamic nature of cancer through its evolution. This assumption led to the exploration of non-tissue sources of tumoral material opening the field of liquid biopsies. Blood, together with body fluids such as urines, or stool, from cancer patients, are analyzed applying the techniques used for the generation of omics data. With blood, this approach would allow to take into account tumor heterogeneity (since the circulating components such as CTCs, ctDNA, or ECVs derive from each cancer clone) in a time dependent manner, resulting in a somehow “real-time” understanding of cancer evolution. Liquid biopsies are beginning nowdays to be applied in many cancer contexts and are at the basis of many clinical trials in oncology.

## Facts

- Oncology has entered the era of Precision Medicine. we still however struggle in capturing the dynamic nature of cancer.
- Liquid biopsies represent a valuable source of information on each individual cancer and can be used to monitor cancer evolution.
- Components of liquid biopsies (CTCs, ctDNA, ctRNA and ECVs) have been proven to correlate with cancer prognosis and cancer biology in many clinical cancer entities (such as breast cancer or neuroblastoma).

## Open questions

- Are liquid biopsies sufficient to capture tumoral evolution?
- How can we discriminate the cancer subclones-derived components of liquid biopsies?
- Will liquid biopsies ever overcome tissue biopsies in the clinical management of cancer?

## Background

Clinical oncology has been relying on the increasing amount of molecular data which can be obtained from single cancer specimens^1^. The molecular profiling of gene mutations for prognostic predictions or for therapy selection can be accounted as a standard approach in many cancer entities since decades. The scientific community moved from a purely histopathologic cancer diagnosis to a molecular-based one, allowing clinicians to develop more accurate and complex prognostic scores, as well as to select better treatment options according to the mutational background of a given neoplasm (as in the case of EGFR or ALK mutations in lung cancer^2^, or the V600E mutation of BRAF in either colorectal cancer^3^ or melanoma, as prominent examples). This approach opened the era of targeted therapies and, to some extent, to precision medicine^4–6^. “Intelligent drugs” designed to specifically target a precise molecular objective, have entered the clinics alongside classic chemotherapy or substituting it^7^. Although this new options generated great therapeutical advances in many cancer contexts (such as the VEGF/VEGFR targeting in kidney cancer^8^ or in hepatocellular carcinoma, or the anti-BCR/Abl rearrangement for the therapy of chronic monocytic leukemia^9^) they still often result in a time limited controlled status of the disease, invariably leading to cancer progression in most cases, without considering primary refractories cancer entities, which are not responsive to given targeted therapies since the beginning^10^. Sometimes, this inevitable failure is partially due to the heterogeneous nature of cancers and to the evolutive pressure that is mediated by cancer treatment itself. Tumor biopsies can give limited information on a single cancer entity, since they cannot account for its intrinsic tumor heterogeneity and, moreover, for its evolution during time, due to their invasive nature which impairs the possibility of their repetition to follow cancer evolution^11–15^.

Great advances in the sequencing technologies are at the roots of the generation of many more accurate molecular data from single cancer specimens. Whole cancer genomes analysis can now be performed at relatively low costs, together with many other “omics”, such as whole transcriptomic or proteomic (which can also be further completed by phospho-proteomics, giving a wide picture on the activation of signaling pathways)^15–24^. These techniques led to the generation of vast amounts of data derived from a single cancer specimen (a diagnostic biopsy or a surgical removal of the disease), therefore determining a molecular deep characterization of a single cancer in a precise and limited time of the disease.

**Fig. 1:**
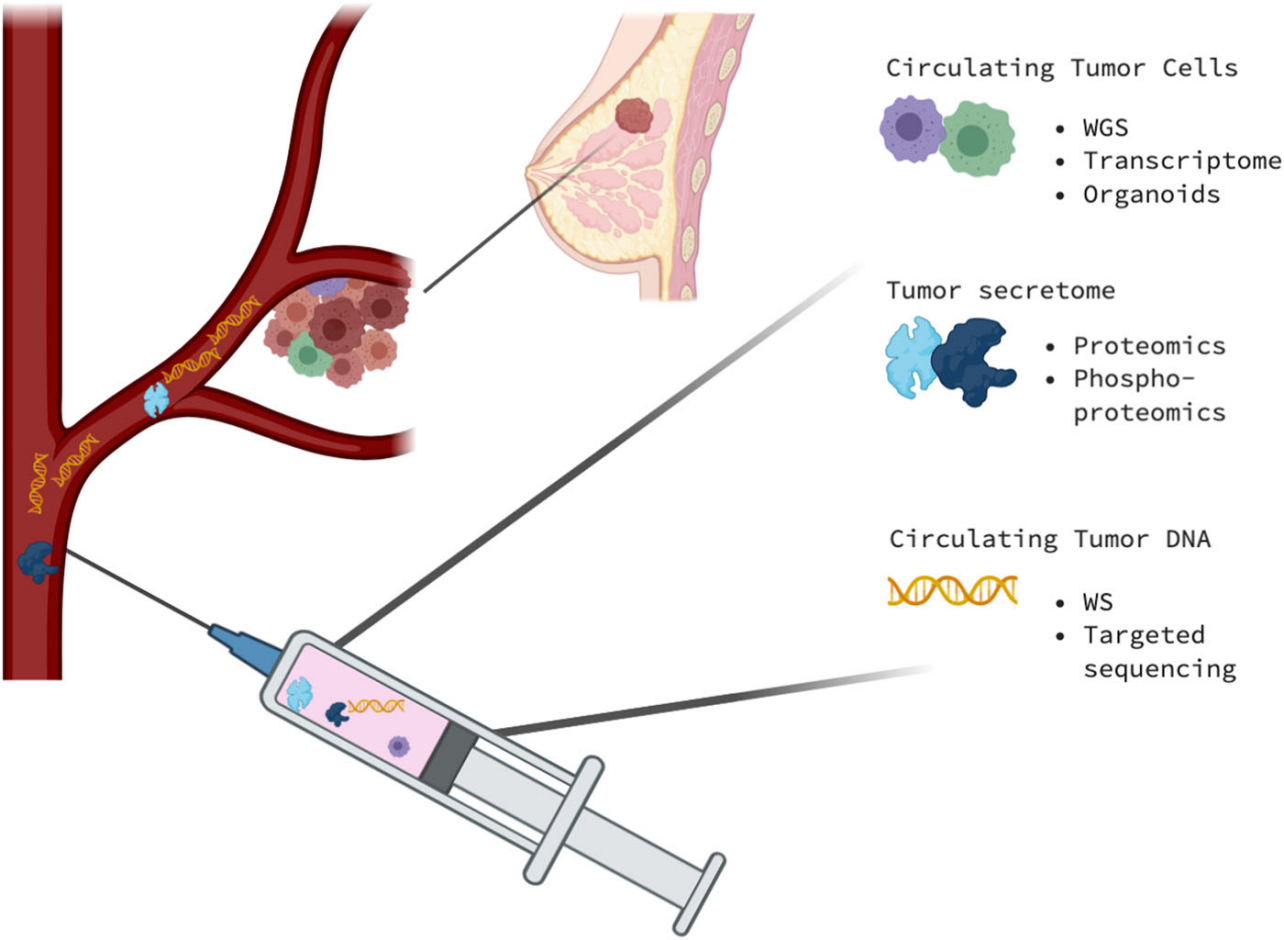
The multiomics approach on liquid biopsies. The information collected from a single blood specimen can reflect the evolution of a single cancer from many biological points of view. Circulating tumor cells (CTCs) highly reflect the complexity of the pathology, especially regarding tumor heterogeneity. Full genomic (whole genome sequencing) and transcriptomic analysis can be applied to CTCs and can be used as well for the growth of cancer organoids. Circulating proteins can also reflect the tumor secretome and can be analyzed through spectrometric approaches such as proteomics and phosho-proteomics. Moreover, circulating tumor DNA can be purified and used for whole or targeted sequencing. Picture created with Biorender.com.

Cancer specimens are not the most ideal source material for capturing the dynamic nature of cancers, since they cannot be repeated during the evolution of the disease due to their invasive nature. Anyway, tissues like blood, have been shown to harbor many biological entities which directly derive from cancer itself and can be exploited as the ideal source for liquid biopsies (Fig. 1)^25–28^.

## Liquid biopsies in cancer

The circulating blood reflects primary and metastatic tumor biology, since tumor cells are more prone than normal cells to release nucleic acids into the bloodstream upon death. Circulating tumor DNA (ctDNA) and well as circulating tumor RNA (ctRNA) can be purified from the plasma of cancer patients, and analyzed through next generation sequencing or targeted sequencing. Both normal and tumoral DNA are circulating and cannot be properly separated form each other but the most abundant source of cell-free DNA is the tumoral one (a mass of 100 g of tumor burden is estimated to release 3.3% of its DNA content) with a 10–100-fold abundancy compared to the normal DNA^29^.

Liquid biopsies can also rely on other aspects of cancer tumorigenesis such as on circulating tumor cells (CTCs) and extra-cellular vesicles (ECVs)^30^. CTCs can be isolated from peripheral blood and discriminated form normal cells using anti-EpCAM or anti-CK or CD45 selection methods. They are extremely rare (<10 cells/ml of blood) and have been isolated from almost all human cancers. CTCs correlate with prognosis and with a metastatic disease status and their clinical role in defining cancer prognosis has been stated by the FDA approval for their detection in breast cancer and prostate cancer^31,32^. CTCs account for tumor heterogeneity since the circulating cells reflect cancer subclones and can be the ideal source for whole genome sequencing and transcriptomic analysis. Moreover, they can also be used to establish organoids cultures, which are proven to be a valuable in vitro reproduction of an individual cancer^33^.

Moving from cells to smaller biological entities, ECVs can be separated from plasma through different methodologies (size exclusion chromatography, affinity purification, and differential ultracentrifugation). As compared to the proteomic profiling of circulating proteins, ECVs show highly enriched exosome proteins and therefore constitute a valuable source of information from a single cancer. Proteins are not the only components of ECVs since tumor DNA and RNA (especially micro-RNA) are also present. Moreover, they are not solely inert tumoral material, since they can function as signal transductor and are highly present in the tumor microenvironment, where they can signal through an autocrine/paracrine pathway or be released in the bloodstream for long distance signaling^34,35^.

Blood is surely the most characterized tissue available for liquid biopsies while many other corporeal fluids are suitable for the detection of tumoral components. Saliva, urines, seminal fluids, tears, and stool have been analyzed to understand their correlation with cancer^36^. Stool in particular have been thoroughly evaluated in the context of gastrointestinal cancers, and proved to be a valuable source of tumoral DNA for pancreatic cancer^37–40^. Stool analysis from a multiomics perspective is also able to integrate information on the role of the microbiome in cancer pathogenesis, especially in the case of colorectal cancer and pancreatic cancer, as well as in other disease entities such as inflammatory intestinal diseases^41–43^. The microbiota creates a complex network that can influence the tumor microenvironment in a very heterogeneous way that relies on the intrinsically heterogeneity of the microbiome itself^44–49^. The microbiome study, also through liquid biopsies, would also grant some other information related to the geography of diseases, among which cancer, since the intestinal bacteria are able to differentiate individuals on the basis of the place they live in^50–54^.

The main advantage of a liquid biopsy approach basically stands with the possibility to capture tumor heterogeneity in a whole (all the tumor subclones release CTCs and ctDNA in the bloodstream) through its evolution, since they are totally not harmful for the patients. The combination of this approach, together with the study of the related omics, has led to promising results in the oncology world.

This review will focus on two disease entities, breast cancer and neuroblastoma, to highlight the state of art in the field.

## Liquid biopsies for breast cancer

**Fig. 2:**
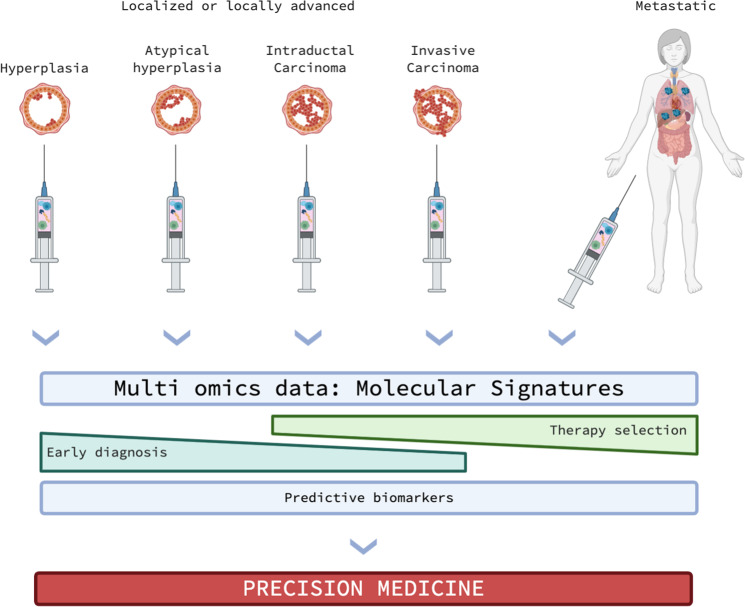
Integrated multiomics approaches in the natural history of breast cancer. Liquid biopsies can capture the complex tumoral genomic and proteomic landscape all along tumor evolution as they can be periodically be repeated due to their relative low harmfulness. This might be of crucial importance and will allow developing early diagnostic tools to detect localized breast cancers, as well as to develop decisional algorithms for the selection of the best therapy at the right moment. All the information acquired will also lead to the identification of precise predictive biomarkers for monitoring the phases of the disease, as well as for the prediction of cancer recurrence. All together, these data will generate e fast and reactive precision medicine approach for the treatment of breast cancer. Picture created with Biorender.com.

Breast cancer represent a striking evidence for the advantage of liquid biopsies use in the everyday medical treatment. The Cellsearch® test has been approved by FDA for the extraction and selection of CTCs from breast cancer patients (but also for prostate cancer) for the determination of their prognosis (Fig. 2)^55,56^.

CTCs arise during early stages of breast cancer as shown on triple negative breast cancer patients (TBNC), where this cell population at diagnosis is quite heterogeneous in terms of expression of hormone receptors (HR), HER2, or EGFR (varying from 24.4% of expression of HR, 20% for HER2 and 40% for EGFR) while only the HER2 positive CTCs prevail after adjuvant treatment^57^. Deeper understanding on breast cancer CTCs comes from the evidence that this cell population expresses both epithelial (keratins, EpCAM, and cadherin 1) and mesenchymal markers (cadherin 2, fibronectin, and serpin peptidase inhibitor), suggesting that they might be subject to the epithelial-to-mesenchymal (EMT) reprogramming. Anyway, the most represented subgroup of CTCs expresses mesenchymal markers therefore confirming that EMT plays a pivotal role in their generation^58^. This is highly remarkable considering that CTCs in breast cancer correlate with metastasis development, and that EMT is highly associated to the development of a metastatic disease from a clinical point of view^59^. CTCs do represent a causative biologic event for the insurgence of metastasis also in the context of brain metastasis, where they show a distinctive “breast cancer brain metastasis gene signature” and can be utilized as predictive biomarkers of this clinical event^60^.

As abovementioned, a liquid biopsy does not only rely on the evaluation of CTCs. The secretion of proteins from tumor cells has been investigated through label-free quantitative proteomics approaches on plasma samples. Tumoral secretomes are highly abundant in plasma since cancer cells are much more prone to shedding and releasing their content into the bloodstream, as compared to healthy cells. The study of secreted proteins through liquid chromatography tandem mass spectrometry combined to RNA sequencing approaches, has been proven to be able to distinguish among breast cancer phenotypes with different prognosis, and proved to be a valuable strategy for outcome prediction^61–64^. Proteogenomics approaches such as the one described were further validated in a larger study conducted on 105 annotated breast cancer genomes, allowing to describe some genomic alterations through a more mechanistic perspective. This is the case of the loss of 5q chromosome, which is a frequent finding in basal-like breast carcinomas, that led to the identification of the loss of CETN3 and SPK1 correlating with higher expression of EGFR^65^.

Proteins are not only secreted in the bloodstream, they can also be incorporated within tumor-derived extracellular vesicles, together with other components such as DNA or RNA. In breast cancer, the role of ECVs has been explored in a mouse model, showing that ECVs target bone marrow CD11+ cells, impairing their differentiation into dendritic cells, leading to the establishment of a pro-metastatic niche, through the production of IL-6^66^. One of the components of tumor derived ECVs is represented by miRNAs. Circulating miRNAs are a highly heterogeneous compartment of the circulating RNA and their evaluation is quite difficult in plasma but, on the counterpart, they are enriched in ECVs. This has been demonstrated on a mouse model of orthotopic xenografts from a breast tumor, showing a high concentration of miR-1246 in ECVs, and confirmed in vesicles isolated from breast cancer patients, correlating with patients’ likelihood of developing recurrencies of the disease^67^. Many other circulating miRNAs and in general noncoding RNAs have been correlated with breast cancer prognosis, miR-34a being significantly downregulated in breast cancer compared to healthy controls^68–71^.

The other abundant tumor-derived circulating component derived from a liquid biopsy is represented by the ctDNA. In the specific case of breast cancer, ctDNA has been correlated to patients’ relapse after surgery and adjuvant treatment, predicting it one to two years before the clinical evidence of the metastatic disease, also correlating with the disease burden^72^. Moreover, ctDNA has been shown to correlate to tumor dynamics in breast cancer patients undergoing surgical or medical therapy^73^, and to be a valuable screening methodology for the selection of breast cancer patients for a specific target drug treatment^74,75^.

Some methodological limitations are still embedded in the liquid biopsies per se. The elements that can be analyzed form a liquid biopsy, especially in the case of ctDNA, still need to be paired to the genomic sequencing of the primary tumor. ctDNA sequencing relies on small circulating fragments which are very diluted among the normal circulating DNA, and therefore represent an impure source of information. Furthermore, in the case of a metastatic disease it is still difficult to assess polyclonal versus monoclonal seeding due to the still ineffective bioinformatics tools available^76^.

## Liquid biopsies in neuroblastoma

Despite relatively rare neuroblastoma (NB) represents a significant clinical problem at pediatric age, accounting for a significant fraction (approximately 10%) of death from children malignancies. Originated from sympathetic nervous system, NB represents the most frequent extracranial tumor in children. Stratification of patients can be conducted on the basis of age at the diagnosis, International Neuroblastoma Staging System (INSS) stage, the MYCN status, Shimada histopathology, and ploidy, defining three well discriminated groups of low, intermediated, and high risk^77^. Genomic abnormalities have indeed been sufficiently defined and linked to the clinical presentation; deletions on chromosomes 1p, 11q, or gains on 17q2,3 are examples of effective prognostic markers of the clinical outcome, despite the molecular basis of their contribution to the pathogenesis are not entirely clear^78–81^. Moreover, neuronal differentiation markers such as p73 have also been associated to the pathogenesis of the disease^82–84^, while the amplification of the proto-oncogene MYCN is another clear predictor of disease severity, therapy resistance and poor clinical outcome. Less than 50% of NB patients with MYCN amplification reach 5-year survival, on contrary of the 90% of non-MYC patients^21,64,85,86^.

Diagnostic procedures for NB largely rely on radiographic body imaging, nuclear medicine examination, evaluation of serum levels of neuron-specific enzymes (enolase and lactate dehydrogenase) or urinary levels of vanillylmandelic acid (VMA) and homovanillic acid (HVA). Sensitivity of these techniques is however low to represent proper early detection methods. Their diagnostic value emerges indeed quite late when the cancer has developed into clinical stage and discrete lumps evident.

Detection of cell free DNA (cfDNA) for NB could improve effectiveness of diagnostic methods and tolerability of medical procedure especially at young age of the patients which are generally involved in this. Detection of MYCN DNA by PCR in the peripheral blood was firstly reported in 2002. Those early data indicated that data the release of MYCN DNA in the blood circulation is an early process in disease, thus offering a potential novel marker for patient follow-up after treatment^87^. Further refinement studies introduced NAGK as a reference gene to quantify MYCN copy number as an MYCN/NAGK (M/N) ratio, providing a more accurate assessment of MYCN status^88^. Subsequent studies have however reduced the optimism, as sensitivity on patients with NB of INSS stages 1 and 2 appeared to be significantly lower than the core biopsy analyses^89,90^. Another putative biomarker is the serum detection of ALK DNA. ALK is a tyrosine kinase receptor subjected to copy number amplification in 25% of NB cases, and some instance mutations in ALK sequence have also been observed. Detection of ALK circulating DNA has therefore been explored to achieve approaches to monitor relapse and predict drug resistance. For example, F1174 and R1275 mutations appear to be effectively detected with high specificity and sensitivity in the peripheral blood of a cohort of NB patients^91^. Additional effort has been placed in development of methods to detect chromosomal variations and DNA methylation in liquid biopsies from NB patients. Good concordance between primary tumor and liquid biopsies has been revealed in detection of several chromosomal variations (11q, 17q, and 1p), it is however still missing a reliable large-scale clinical trial reporting substantial evidence to support shift of diagnostic procedures to detection of cfDNA. At the present, clinical practise therefore relies on histopathology and molecular characterization of tumor tissue^63,92,93^. However, unfeasibility of multiple resections, irregular interval between morbidity and acquisition of diagnostic material and ambiguous interpretations of pathology samples are just some of the substantial drawbacks of these classic procedures.

## Conclusions

Oncology has undoubtedly entered the precision medicine era with the technological ability to characterize a single cancer from multiomics approaches. This possibility can be exploited also thanks to some major advances in artificial intelligence algorithms, that are essential for deciphering much of the information embedded in the omics data. Anyhow, this huge amount of biologic details does not readily and dynamically translate into the clinical practice, since it is usually performed on the cancer tissue itself, that is normally taken one (or a very few times) during individual cancer history. This limitation does not only regard time, but also capturing tumor heterogeneity. The improvement of our understanding of cancer biology, with the discovery that tumoral material circulates in the bloodstream in form of CTCs, ctDNA, or ctRNA, EVCs, has given the opportunity to conceive the idea that blood itself might represent a cancer biopsy per se. Under this light, liquid biopsies can overcome many limitations of tissue biopsies and could capture tumor heterogeneity in a whole, thanks to the improvement of both omics technologies and the associated artificial intelligence elaboration of the data, but mostly can capture tumor evolution without being invasive to the patients. This will be soon translated into a more precise prognostic evaluation and a best treatment selection designed on a single patient’s disease during its evolution, leading to a true precision medicine approach.
